# RPLC- and HILIC-based non-targeted metabolomics workflow for blood microsamples

**DOI:** 10.1007/s11306-026-02402-y

**Published:** 2026-02-09

**Authors:** Pauline Couacault, Michael Witting

**Affiliations:** 1 Metabolomics and Proteomics Core, Helmholtz Zentrum München, Ingostädter Landstraße 1, 85764 Neuherberg, Germany; 2 Chair of Analytical Food Chemistry, TUM School of Life Sciences, Technical University of Munich, Maximus-von-Imhof-Forum 2, Weihenstephan, 85354 Freising, Germany

**Keywords:** Non-targeted metabolomics, Blood microsampling, HILIC-MS, RPLC-MS, VAMS, DBS

## Abstract

**Introduction:**

Blood microsampling (BμS) has emerged as an alternative to invasive sampling methods, including blood and plasma sampling. Several studies have shown that BμS are suitable alternatives for analyzing endogenous metabolites and for metabolomics applications. Dried blood spots (DBS) have long been used for clinical applications, particularly for newborn screening. New quantitative BμS have emerged, including volumetric absorptive microsampling (VAMS).

**Objectives:**

We aimed to develop an extraction protocol from BµS for non-targeted metabolomics analysis using a reversed-phase liquid chromatography/mass spectrometry (RPLC-MS) method for the mid- to non-polar metabolome and a hydrophilic interaction chromatography/mass spectrometry (HILIC-MS) method for the polar metabolome, based on existing protocols from the literature. To improve coverage, two new HILIC-MS methods have been developed.

**Methods:**

We used an in-house RPLC-MS method for the analysis of mid- to non-polar metabolites. Two new HILIC-MS/MS methods were developed using 73 chemical reference standards of polar metabolites from various classes. To optimize extraction, five procedures were investigated and compared to identify the most appropriate protocol for extracting metabolites from BµS for non-targeted metabolomics analysis. The final workflow was optimized on both DBS and VAMS.

**Results and conclusion:**

We developed and optimized a 15-minute HILIC-MS method that included column re-equilibration. Our experiments showed that using a 20% H_2_O/80% MeOH (v/v) mixture for extraction, with sample rehydration, is a good compromise for detecting many metabolite features. Our extraction and LC-MS methodology covered metabolites from many pathways, including amino acids, acylcarnitines, and bile acids.

**Supplementary Information:**

The online version contains supplementary material available at 10.1007/s11306-026-02402-y.

## Introduction

Metabolomics — the systematic analysis of metabolites in a given system — has garnered considerable interest in the (bio)medical community as a potential extension of analytical approaches used in clinical chemistry (Marchev et al., 2021; Skogvold et al., 2025). Likewise, lipidomics — the analysis of a sample’s lipid complement, analogous to metabolomics — is gaining considerable attention (Salihovic et al., 2023). Metabolomics and lipidomics analyses in humans have typically been conducted on clinically established specimens, such as plasma or serum. However, there has been a recent shift towards the use of alternative sampling methods for health and disease monitoring. Dried blood spots (DBS), for example, were originally described in the 1960 s by Robert Guthrie to measure phenylalanine levels in blood and detect phenylketonuria in newborns (Guthrie, 1961; Guthrie & Susi, 1963). Nowadays, newborn screening, primarily based on tandem mass spectrometry (MS/MS), is performed in more than 140 countries worldwide, covering inborn errors of metabolism involving organic acids, amino acids, fatty acid oxidation, and hemoglobin (Therrell et al., 2024). DBS is also used in conjunction with mass spectrometry (MS) for therapeutic drug monitoring (Zailani & Ho, 2023), anti-doping (Okano et al., 2022; “Paris 2024 Anti-Doping Guide,” 2024; Wang et al., 2022), toxicology analysis (Ververi et al., 2023), or exposure detection (Jacobson et al., 2023).

Blood microsamples (BµS), such as DBS, are suitable alternatives to venous blood, plasma, or other invasive sampling methods for profiling human health, especially in vulnerable groups, including infants, the elderly, or those with illnesses. Indeed, they are non-invasive since there is no need for phlebotomist or needle and need a low volume of blood (< 100 µL) (Thangavelu et al., 2023); they are simple to use and can be used for at home-sampling (Spooner et al., 2020); they are easy to store and most analytes show a good stability for long-term storage, which helps build blood biobanks (Ottosson et al., 2023). For these reasons, there has been recent interest in using BµS in combination with metabolomics and lipidomics for health monitoring, rather than classical plasma or serum samples (Ferreira et al., 2022; Nys et al., 2017).

However, DBS is not a quantitative collection technique. Indeed, each drop of blood has a different volume, and each blood type has its own hematocrit, the percentage of red cells. Blood viscosity is affected by hematocrit, which in turn influences the size, color, and homogeneity of the blood spot (O’Mara et al., 2011). All these factors can make the accurate quantification of analytes more challenging. To overcome this limitation, volumetric BµS that can collect a fixed volume of blood were created. Volumetric absorptive microsampling (VAMS) (de Sá e Silva et al., 2023; Spooner et al., 2015) utilizes a polymeric tip that can absorb a specific volume (10, 20, or 30 µL) of blood through capillary action.

Non-targeted metabolomics is primarily performed using reversed-phase liquid chromatography (RPLC) (Gertsman & Barshop, 2018; Gika et al., 2023), and analysis on BµS is no exception (Couacault et al., 2024). However, this method cannot detect polar metabolites, such as amino acids and sugars, which can be detected by hydrophilic interaction chromatography (HILIC). The combination of these two techniques enables a broader coverage of metabolites, particularly in non-targeted analysis.

In this study, we aimed to develop an extraction protocol for BµS to facilitate non-targeted metabolomics analysis using RPLC-MS. Since BµS contain blood cells as well, many more intracellular and polar metabolites are expected to be present in these samples. Therefore, we also optimized a HILIC-MS method for the analysis of the polar metabolome. With run times of 15 min per injection, including ionization, and column re-equilibration, the method provides sufficient resolution of important metabolites at a reasonable runtime. Using a universal extraction method and combining RPLC-MS and HILIC-MS will enable the widespread application of BµS and metabolomics in the future, covering a large metabolite space relevant for multiple applications.

## Materials and methods

### Reagents and materials

All chemicals and solvents used were of LC-MS grade or analytical grade. Seventy-three (73) polar metabolite standards were purchased for the HILIC-MS method development. A detailed list of metabolite standards, abbreviations, and manufacturers is available in the Supplementary Table S1. Water (H_2_O), acetonitrile (ACN), methanol (MeOH), and ethanol (EtOH) were purchased from Merck (Darmstadt, Germany). Ammonium formate (AmF) (10 M stock solution), ammonium acetate (AmAc), ammonium bicarbonate (AmBc), and ammonium hydroxide (28.0%−30.0% NH_3_ basis) were purchased from Sigma-Aldrich (Sigma-Aldrich Chemie GmbH, Taufkirchen, Germany). Formic acid was purchased from Fischer Chemical (Fisher Scientific GmbH, Schwerte, Germany). Purified water (18.2 MΩ) used for extraction was obtained from a Milli-Q integral water purification system (Billerica, MA, United States of America). ESI Positive Calibration Solution X500B and ESI Negative Calibration Solution X500 were purchased from Sciex (Sciex Deutschland GmbH, Darmstadt, Germany).

### Sample preparation

The use of human samples was approved by the Research Ethics Committee of Aristotle University of Thessaloniki (#306272/2022). The blood collection was undertaken after written informed consent. All experiments were performed in accordance with the guidelines of the Declaration of Helsinki. Twenty-microliter (20 µL) Mitra devices were acquired from Neoteryx (Torrance, CA). Whatman^®^ 903 protein saver cards, each containing five collection spots, were obtained from Sigma-Aldrich (St. Louis, MI, USA). Antecubital venipuncture blood was collected by a trained phlebotomist from healthy, overnight-fasted individuals in EDTA tubes. BµS were collected for each individual. For DBS collection, the blood was pipetted onto each spot of the card, creating 50 or 20 µL spots depending on the experiment. For VAMS collection, the tip was partially dipped in the EDTA tube for approximately 6 s. The BµS samples were left to dry at room temperature for 3 h before storage at − 80 °C in desiccant pouches until analysis (Thaitumu et al., 2025).

#### Extraction solvent optimization

50 µL DBS (Whatman^®^ 903) were prepared at Aristotle University of Thessaloniki (Thessaloniki, Greece) (Thaitumu et al., 2025) and stored at −80 °C prior to analysis. Four extraction procedures were tested with five replicates each: 20% H_2_O/80% MeOH (v/v), 50% H_2_O/50% MeOH (v/v), 20% H_2_O/80% ACN (v/v), and 50% MeOH/50% EtOH (v/v). To preserve metabolite integrity and avoid potential metabolite degradation, extractions were performed at cold temperatures.

The samples were thawed on ice before sample preparation. 1 mL of the respective solvent mixture was added to the whole cut-out DBS. Then, the samples were sonicated in an ice bath for 10 min and centrifuged at 14,000 rpm for 10 min in a cold room (4–8 °C). Supernatants were collected and divided into two equal aliquots (RPLC + and RPLC-). They were then dried under nitrogen and stored at −80 °C prior to MS analysis. On the day of the analysis, aliquots for RPLC-MS were reconstituted in 40 µL of 80% H_2_O/20% ACN (v/v), sonicated for 5 min, and centrifuged for 5 min at 2,650 rpm.

#### Extraction optimization

20 µL DBS (Whatman^®^ 903) and 20 µL VAMS (Mitra^®^) were prepared at Aristotle University of Thessaloniki (Thessaloniki, Greece) (Thaitumu et al., 2025) and stored at −80 °C prior to analysis. Two extraction procedures were tested, each with four replicates: 20% H_2_O/80% MeOH (v/v) with sample rehydration and 20% H_2_O/80% MeOH (v/v) without sample rehydration. To preserve metabolite integrity and avoid potential metabolite degradation, extractions were performed at cold temperatures.

The samples were thawed on ice before sample preparation. For the experiment without sample rehydration, 400 µL of 20% H_2_O/80% MeOH (v/v) was added directly to the whole BµS. Then, the samples were sonicated in an ice bath for 10 min and centrifuged at 14,000 rpm for 10 min in a cold room (4–8 °C). For sample rehydration experiments, 80 µL of water was first added to the sample. Samples were sonicated in an ice bath for 10 min and centrifuged at 14,000 rpm for 10 min in a cold room (4–8 °C). 320 µL of MeOH was added to the BµS; then, the samples were sonicated in an ice bath for 10 min and centrifuged at 14,000 rpm for 10 min in a cold room (4–8 °C). In total, 400 µL of solvent was added to the devices. Supernatants were collected and divided into four equal aliquots (RPLC+, RPLC-, HILIC+, HILIC-). These aliquots were then dried under nitrogen and stored at −80 °C prior to MS analysis. On the day of the analysis, aliquots for RPLC-MS were reconstituted in 40 µL of 80% H_2_O/20% ACN (v/v) and aliquots for HILIC-MS were reconstituted in 40 µL of 10% H_2_O/90% ACN (v/v), sonicated for 5 min, and centrifuged for 5 min at 2,650 rpm.

#### Final sample preparation protocol

The final extraction protocol, based on previous tests, is described here. To preserve metabolite integrity and avoid potential metabolite degradation, extractions were performed at cold temperatures. BµS samples of 20 µL were thawed on ice. Samples were mixed with 80 µL of water for rehydration. Samples were sonicated in an ice bath for 10 min and centrifuged for 10 min at 14,000 rpm in a cold room (4–8 °C). 320 µL of MeOH with internal standards was added to the rehydrated samples. Samples were sonicated a second time in an ice bath for 10 min and then centrifuged for 10 min at 14,000 rpm in a cold room (4–8 °C). Supernatants were collected and divided into four equal aliquots (RPLC+, RPLC-, HILIC+, HILIC-). These aliquots were then dried under nitrogen and stored at −80 °C prior to MS analysis. On the day of the analysis, aliquots for RPLC-MS were reconstituted in 40 µL of 80% H_2_O/20% ACN (v/v) and aliquots for HILIC-MS were reconstituted in 40 µL of 10% H_2_O/90% ACN (v/v), sonicated for 5 min, and centrifuged for 5 min at 2,650 rpm.

### LC-MS methods

#### HILIC method optimization

For testing and screening of different HILIC columns and separation conditions, an in-house created solution of 73 polar metabolite reference standards was used. A detailed list of metabolite standards, abbreviations, and manufacturers is available in the Supplementary Table S1. All standards were dissolved at appropriate concentrations in the adequate solvent, and the final concentration in the full mixture was 10 ppm in 10% H_2_O/90% ACN (v/v). For isomers, both single mixture solution – to check the single compound RT – and the full in-house standard solution – to check the isomers separation – were injected.

Three different HILIC columns, one with an amide stationary phase and two with zwitterionic stationary phases, were tested: Waters ACQUITY UPLC BEH Amide (100 mm x 2.1 mm, 1.7 μm), Waters Atlantis Premier BEH Z-HILIC (100 mm x 2.1 mm, 1.7 μm), and Agilent InfinityLab Poroshell 120 HILIC-Z (100 mm x 2.1 mm, 2.7 μm). All columns were compared using the same eluents and the same gradient. In positive ionization mode, eluent A consisted of 10 mM AmF in water (pH 2), and eluent B consisted of 10 mM AmF in ACN (pH 2) (Dai & Hsiao, 2019; Walter et al., 2021). In negative ionization mode, eluent A consisted of 10 mM AmBc in water (pH 9), and eluent B consisted of 10 mM AmBc in ACN (pH 9) (Smith et al., 2022; Walter et al., 2021). The binary gradient for both ionization modes was as follows: 0 min, 90% B; 2.5 min, 90% B; 10 min, 10% B; 14 min, 10% B; 15 min, 90% B; 20 min, 90% B. The flow rate was 0.5 mL/min with the column temperature set at 40 °C. Mobile phase optimization was performed by comparing multiple previously used eluent compositions. For positive ionization mode, the recommended buffer system is AmF with low pH (Dai & Hsiao, 2019; Walter et al., 2021). Since the results from the stationary-phase optimization were sufficient, no further optimization was performed. For negative ionization mode, the two recommended buffer systems are AmAc (Dai & Hsiao, 2019; Hsiao et al., 2019; Smith et al., 2022; Yannell et al., 2023) and AmBc (Smith et al., 2022; Walter et al., 2021) with high pH. Eluent A consisted of 10 mM AmBc or AmAc in water (pH 9), and eluent B consisted of 10 mM AmBc or AmAc in 10% H_2_O/90% ACN (v/v) (pH 9).

#### Final HILIC-MS/MS method

Samples were analyzed using an Agilent 1290 Infinity II Bio (Agilent Technologies Deutschland GmbH, Böblingen, Germany) UHPLC system coupled with a Sciex ZenoTOF 7600 (Sciex Deutschland GmbH, Darmstadt, Germany). MS analyses were performed in positive and negative ionization mode with a mass range of 50–1500 m/z. Mass spectra were acquired in data-dependent mode, and the twelve highest MS^1^ ions were chosen for MS/MS. The MS parameters are provided in Supplementary Table S2. The HILIC positive method used an Agilent InfinityLab Poroshell 120 HILIC-Z column (100 mm x 2.1 mm, 2.7 μm, Agilent Technologies Deutschland GmbH, Böblingen, Germany); eluent A consisted of 10 mM AmF and 0.1% of formic acid in water; eluent B consisted of 10 mM AmF and 0.1% formic acid in 10% H_2_O/90% ACN (v/v). The HILIC negative method used a Waters Atlantis Premier BEH Z-HILIC column (100 mm x 2.1 mm, 1.7 μm, Waters GmbH, Eschborn, Germany); eluant A consisted of 10 mM AmAc and 0.05% ammonium hydroxide 28% in water (pH9); eluent B consisted of 10 mM AmAc and 0.05% ammonium hydroxide 28% in 10% H_2_O/90% ACN (v/v) (pH9). Eluents for HILIC analysis were prepared in Nalgene^®^ FEP bottles (Sigma-Aldrich Chemie GmbH, Taufkirchen, Germany). Detailed LC parameters are provided in the Supplementary Tables S3 and S4.

#### RPLC-MS/MS method

No optimization of the RPLC-MS method was performed, as a previously validated in-house method used for blood-derived samples was available (Artati et al., 2025). Samples were analyzed using a Sciex ExionLC AD (Sciex Deutschland GmbH, Darmstadt, Germany) UHPLC system coupled with a Sciex ZenoTOF 7600 (Sciex Deutschland GmbH, Darmstadt, Germany) for RPLC-MS analysis. MS analyses were performed in positive and negative ionization mode with a mass range of 50–1500 m/z. Mass spectra were acquired in data-dependent mode, and the twelve highest MS^1^ ions were chosen for MS/MS. The MS parameters are provided in Supplementary Table S2. The RPLC method utilized a Phenomenex Kinetex C18 column (100 mm x 2.1 mm, 1.7 μm, Phenomenex Inc., Aschaffenburg, Germany). Eluent A consisted of water with 0.1% formic acid, and eluent B consisted of ACN with 0.1% formic acid. Detailed LC parameters are provided in the Supplementary Tables S3 and S4.

### Data processing and evaluation

Targeted data processing of raw data.wiff files, including internal and reference standards detection, and instrument performance check, was performed with Sciex OS software (Sciex Deutschland GmbH, Darmstadt, Germany). Non-targeted data processing of raw data.wiff files, including peak picking, peak alignment, and compound annotation, was performed with mzmine 4.5 (Schmid et al., 2023). Statistics and visualization were performed on R 4.5.1 (R Core Team, 2025).

Comparison of features across different extraction protocols was performed based on feature counts and on the ratio of the average feature area within each extraction method to the overall average across all extraction methods. This factor has been calculated for each feature in each analytical mode. A ratio > 1 indicates that this feature has been detected at higher relative quantities in this specific extraction method than in the average across all extraction methods.

Evaluation of column and solvent selection is based on the number of standards detected, chromatographic peak shape and intensity, isomer separation, and complementary aspects of both ionization modes. Detected peaks were manually graded as “good” if their height was higher than 1e4 counts, and their width at 5% height was lower than 0.5 min; “broad” if their width at 5% height was higher than 1 min; “low intensity” if their height was lower than 1e3 counts; other detected peaks were marked as “acceptable”.

## Results and discussion

### Optimization of the extraction solvent

Multiple protocols for the extraction and measurement of BµS have been published (Bossi et al., 2025; Volani et al., 2017). While extraction protocols generally differ only slightly, it is important to optimize and compare them based on the specific analytical equipment available and the goals of the study. To establish our workflow, we first aimed to develop an extraction protocol for non-targeted metabolomics of BµS compatible with RPLC-MS, since this is the most commonly used analytical platform in metabolomics, especially for blood-derived samples (Couacault et al., 2024; Gertsman & Barshop, 2018; Gika et al., 2023). The extraction procedure needed to be quick, simple, and effective, capable of extracting a wide range of metabolites for optimal use in future studies. To preserve metabolite integrity and avoid potential metabolite degradation, the extractions were performed at cold temperatures.

The first step was to identify the optimal extraction solvent that balances metabolite coverage and ease of use. An experiment was conducted based on literature research identifying commonly used extraction solvents for DBS (Couacault et al., 2024). Four solvent combinations were tested: 20% H_2_O/80% MeOH (v/v), 50% H_2_O/50% MeOH (v/v), 20% H_2_O/80% ACN (v/v), and 50% MeOH/50% EtOH (v/v). 1 mL of solvent was added to the total cut-out of the DBS samples, containing 50 µL of whole blood each. Then, the samples were sonicated in an ice bath for 10 min and centrifuged at 14,000 rpm for 10 min in a cold room (4–8 °C). Supernatants were collected and divided into two equal aliquots (RPLC + and RPLC-), dried under nitrogen, and stored at −80 °C prior to LC-MS analysis. On the day of the LC-MS analysis, aliquots for RPLC-MS were reconstituted in 40 µL of 80% H_2_O/20% ACN (v/v), sonicated for 5 min, and centrifuged for 5 min at 2,650 rpm.

**Fig. 1:**
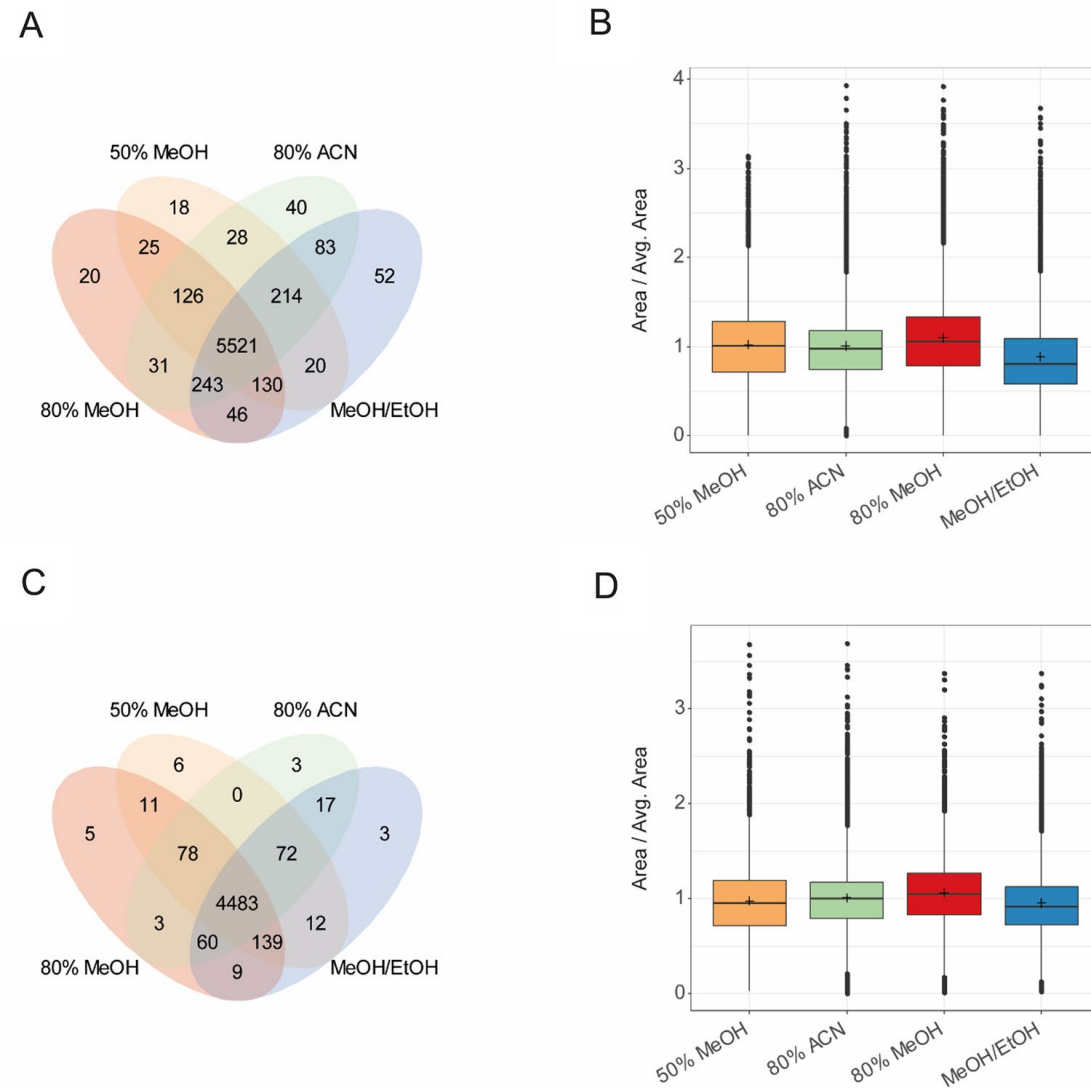
**A** Venn diagram showing the overlap of features between the different extraction solvents for RPLC positive ionization mode. **B** Box plot of peak’s area over average peak’s area ratio for all tested extraction solvents for RPLC positive ionization mode. **C** Venn diagram showing the overlap of features between the different extraction solvents for RPLC negative ionization mode. **D** Box plot of peak’s area over average peak’s area ratio for all tested extraction solvents for RPLC negative ionization mode

Data were processed with mzmine 4.5 and evaluated based on the number of detected features and their overlap between the different solvent mixtures (Fig. 1A and C). Generally, most features are present in all solvent mixtures and yield very similar numbers in both positive and negative ionization modes. On average, most features were detected with the 20% H_2_O/80% MeOH (v/v) solvent mixture. These results align with the existing literature, which indicates that this solvent mixture is a suitable compromise in various matrices (Artati et al., 2025; Gika & Theodoridis, 2011; Ottosson et al., 2023; Ser et al., 2015; Vuckovic, 2019; Wishart et al., 2008). As an additional differentiator between the solvent mixtures, we compared intensities of detected features (Fig. 1B and D). We have calculated a factor for the peak’s area of a feature in the respective solvent mixtures, relative to the average peak’s area across all solvent mixtures. 20% H_2_O/80% MeOH (v/v) showed slightly higher values compared to the other solvents and was therefore selected for further experiments.

### HILIC chromatography optimization

Since RPLC-MS can only analyze mid- to non-polar metabolites, we may potentially miss important polar metabolites that elute within the void volume. However, analysis of the void volume is unreliable due to the lack of chromatographic separation suppression caused by heavy co-elution. HILIC-MS has become a valuable alternative for analyzing polar metabolites. Indeed, DBS samples contain blood cells, so a higher number of polar, intracellular metabolites is expected to be detected. To separate and detect polar metabolites from DBS and other BµS, a non-targeted HILIC-MS method was developed.

For testing and screening of different HILIC columns and separation conditions, an in-house solution containing 73 polar metabolite reference standards from various polar metabolite classes, including amino acids, carnitines, organic acids, and sugar phosphates, was used. Metabolites expected to be present in higher amounts in blood cells, such as nucleotides, were included in the optimization, as they are likely to be present in BµS due to the use of whole blood, which also contains cellular content. Additionally, we have included other polar metabolites, such as CoA and short-chain acyl-CoAs, to test whether the method can be of value for other sample matrices, e.g., tissues and cells. We compared the number of detected molecules, chromatographic peak shape, peak intensity, and isomer separation for each candidate column. For RPLC, generic eluents are often used for both positive and negative ionization modes, and no ionization mode-specific optimization is performed; however, this is required for HILIC to achieve optimal results. Since polar metabolites are structurally very diverse (e.g. positively charged molecules such as amines, zwitterionic substances such as amino acids, or negatively charged molecules such as organic acids) (Dai & Hsiao, 2019; Gallart-Ayala et al., 2018), we chose to develop two different LC methods with different pH values for the different ionization modes to maximize our coverage.

**Fig. 2:**
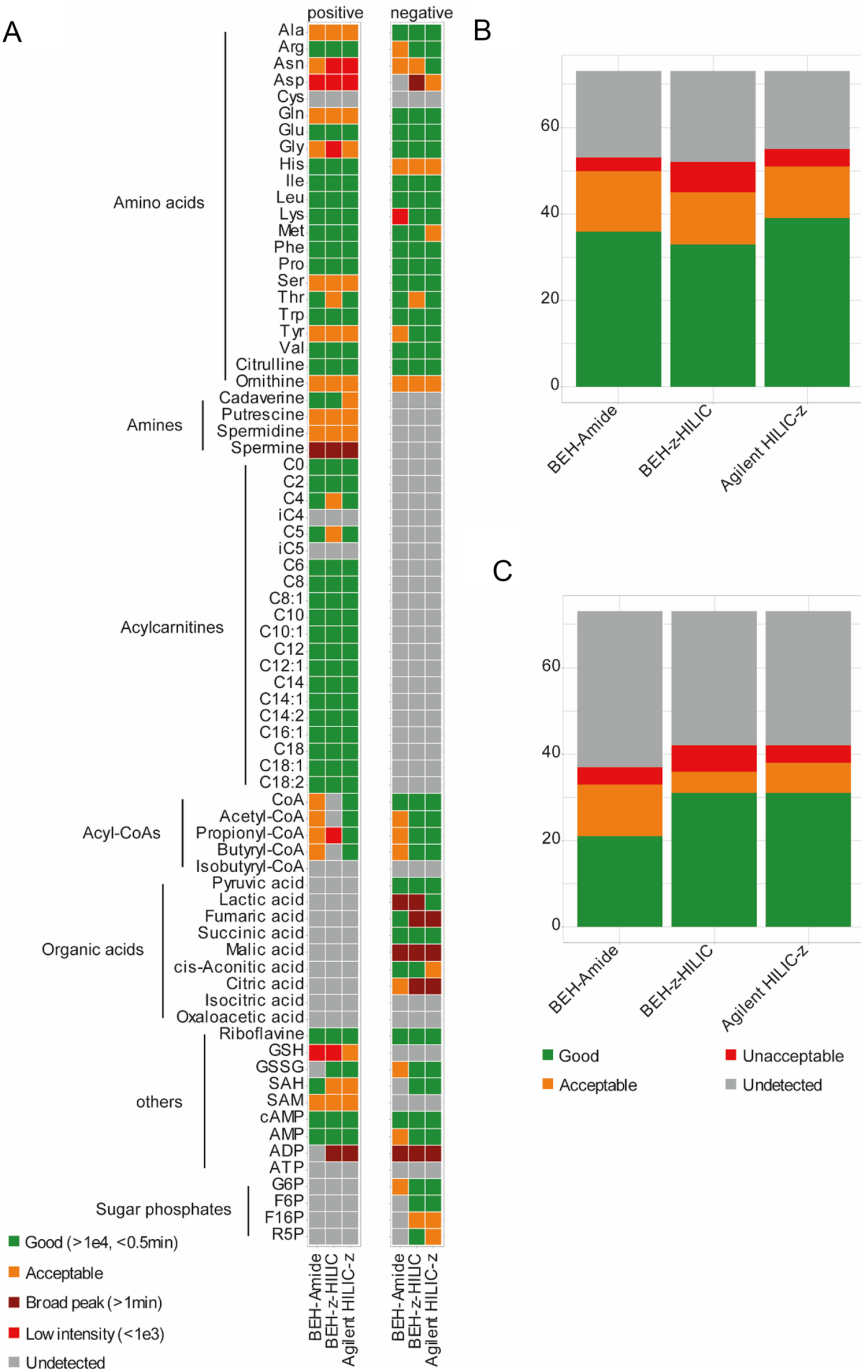
**A** Individual performance of candidate columns in positive and negative ionization mode. Scoring is based on peak shapes and intensities. **B** Bar plots representing the number of detected standards analyzed by different candidate columns C: Bar plots representing the number of detected standards analyzed by different candidate columns in negative ionization mode

In total, three different HILIC columns, one with an amide stationary phase and two with zwitterionic stationary phases, were tested: Waters ACQUITY UPLC BEH Amide (100 mm x 2.1 mm, 1.7 μm), Waters Atlantis Premier BEH Z-HILIC (100 mm x 2.1 mm, 1.7 μm), and Agilent InfinityLab Poroshell 120 HILIC-Z (100 mm x 2.1 mm, 2.7 μm). All columns were compared using the same eluents and the same gradient as those used in the initial optimization step. In positive ionization mode, eluent A consisted of 10 mM AmF in water (pH 2), and eluent B consisted of 10 mM AmF in ACN (pH 2) (Dai & Hsiao, 2019; Walter et al., 2021). In negative ionization mode, eluent A consisted of 10 mM AmBc in water (pH 9), and eluent B consisted of 10 mM AmBc in ACN (pH 9) (Smith et al., 2022; Walter et al., 2021). AmBc was chosen instead of AmAc for negative ionization mode, as several studies have shown that AmBc is beneficial for anionic compound detection (Nakatani et al., 2022; Nilsson et al., 2024; Zhang et al., 2024). The binary gradient for both ionization modes was as follows: 0 min, 90% B; 2.5 min, 90% B; 10 min, 10% B; 14 min, 10% B; 15 min, 90% B; 20 min, 90% B. The flow rate was 0.5 mL/min with the column temperature set at 40 °C. The best column for each ionization mode was selected based on the number of standards detected, the chromatographic peak shape, peak intensity, isomer separation, and complementary aspects of both ionization modes. A summary of the results is presented in Fig. 2A-C. Detected peaks were manually graded as “good” (green) if their height was higher than 1e4 counts, and their width at 5% height was lower than 0.5 min; “broad” (dark red) if their width at 5% height was higher than 1 min; “low intensity” (light red) if their height was lower than 1e3 counts; other detected peaks were marked as “acceptable” (orange).

In positive ionization mode, most amino acids could be detected by all columns. However, for the Agilent HILIC-Z, we observed a high background noise (> 4e4) in the EIC of phenylalanine. Spermine and spermidine showed broader peaks (respectively, > 3 min and 1 min) on all columns. All columns were able to detect acyl carnitines, but their peak shapes were better with Agilent HILIC-Z and BEH-Amide; indeed, with BEH-Z-HILIC, peaks were broader, and long-chain acyl carnitines (> 10 carbon units) sometimes displayed double peaks. CoA and its derivatives were correctly detected only by Agilent HILIC-Z; with BEH-Amide, the peaks were broader and less well-defined. Generally, nucleotides and derivatives, as well as glutathione and related molecules, showed better detection with zwitterionic columns (BEH-Z-HILIC and Agilent HILIC-Z).

In negative ionization mode, amino acids showed broader peaks with Agilent HILIC-Z, which the employed buffer can explain. HILIC-Z is typically used with AmAc (Dai & Hsiao, 2019; Hsiao et al., 2019; Yannell et al., 2023), whereas BEH-Z-HILIC is used with AmBc (Smith et al., 2022; Walter et al., 2021). Overall, most amino acids showed better peak shape in positive ionization mode compared to negative ionization mode. Indeed, most peaks were broader; glycine showed multiple peaks, and those of histidine and ornithine were less defined in negative-ion mode. But unlike in positive ionization mode, phenylalanine was detected with no background noise. Aspartic acid peaks were less defined, but they were more intense and sharper with Agilent HILIC-Z than BEH-Z-HILIC. All columns could detect CoA and its derivatives; however, they showed broader peaks with BEH-Amide. Most organic acids didn’t show good peak shape across all columns (multiple, less-defined peaks and high background noise). Similar to the positive ionization mode, nucleotides, glutathione, and related molecules showed better detection with zwitterionic columns (BEH-Z-HILIC and Agilent HILIC-Z); however, ADP exhibited broad, less-defined peaks. Sugar phosphates showed better peak shape with BEH-Z-HILIC.

**Fig. 3:**
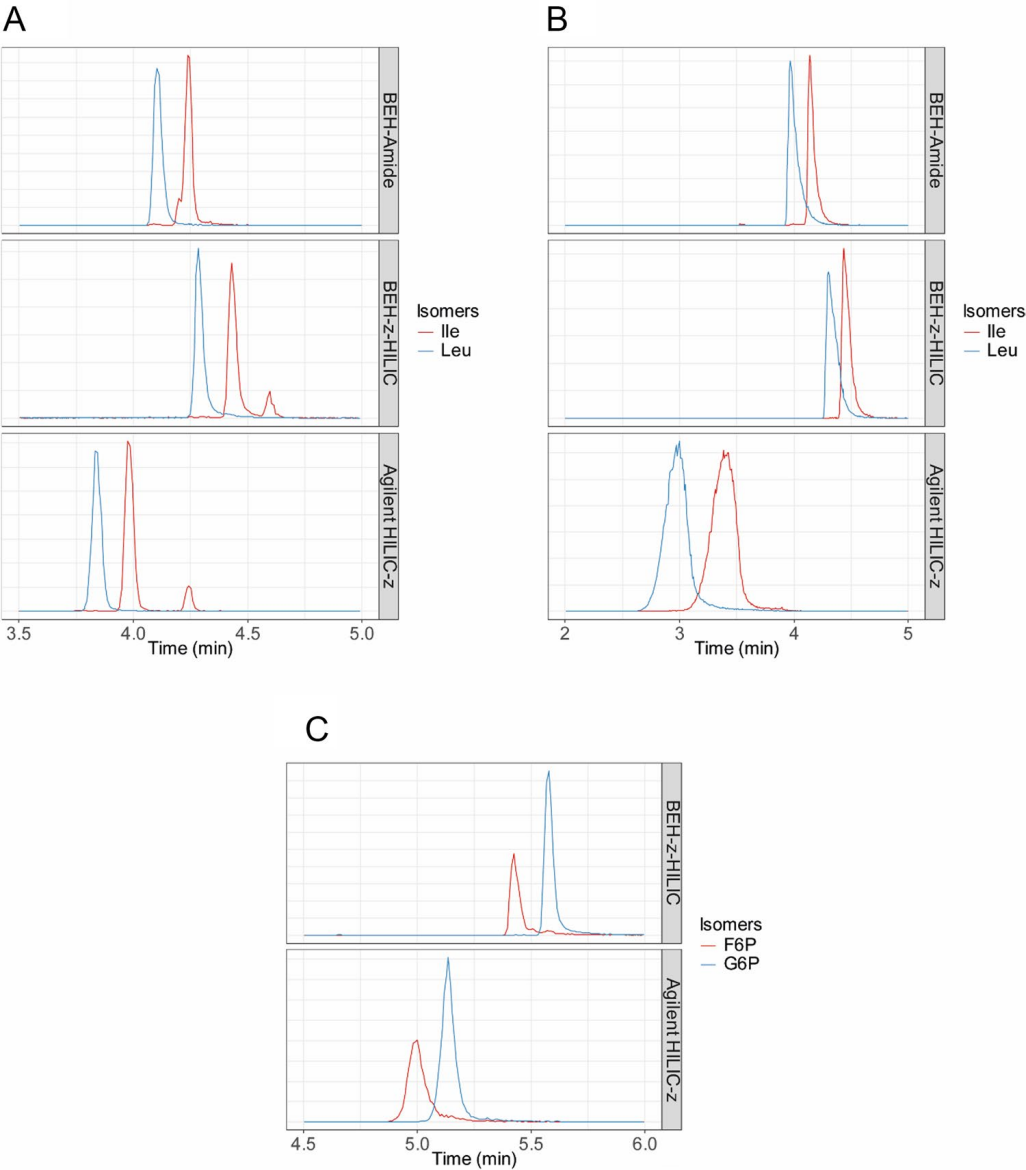
**A** Extracted ion chromatogram of the isomeric pair leucine/isoleucine in positive ionization mode. **B** Extracted ion chromatogram of the isomeric pair leucine/isoleucine in negative ionization mode. **C** Extracted ion chromatogram of the isomeric pair Glucose-6-phosphate/Fructose-6-phosphate in negative ionization mode

Not only retention, but also separation of isomeric substances is essential. We therefore evaluate the performance of each column in separating known isomers from our standard mixtures, such as leucine/isoleucine or G6P/F6P (Fig. 3). All columns showed a good separation of leucine and isoleucine. Since F6P was not detected with BEH-Amide, only the zwitterionic columns were able to separate F6P from G6P. None of the employed columns was able to separate citric acid/isocitric acid, butyryl-CoA/isobutyryl-CoA, or the carnitine isomers (C4/iC4 and C5/iC5) under the current conditions. None of the employed columns was able to detect cysteine, oxaloacetic acid, and ATP under the current conditions.

In positive ionization mode, Agilent HILIC-Z showed better metabolite coverage. In negative ionization mode, both zwitterionic columns (BEH-Z-HILIC and Agilent HILIC-Z) showed better results and were complementary to the chosen positive ionization mode column. These two columns were then compared for later optimization of mobile phases.

**Fig. 4:**
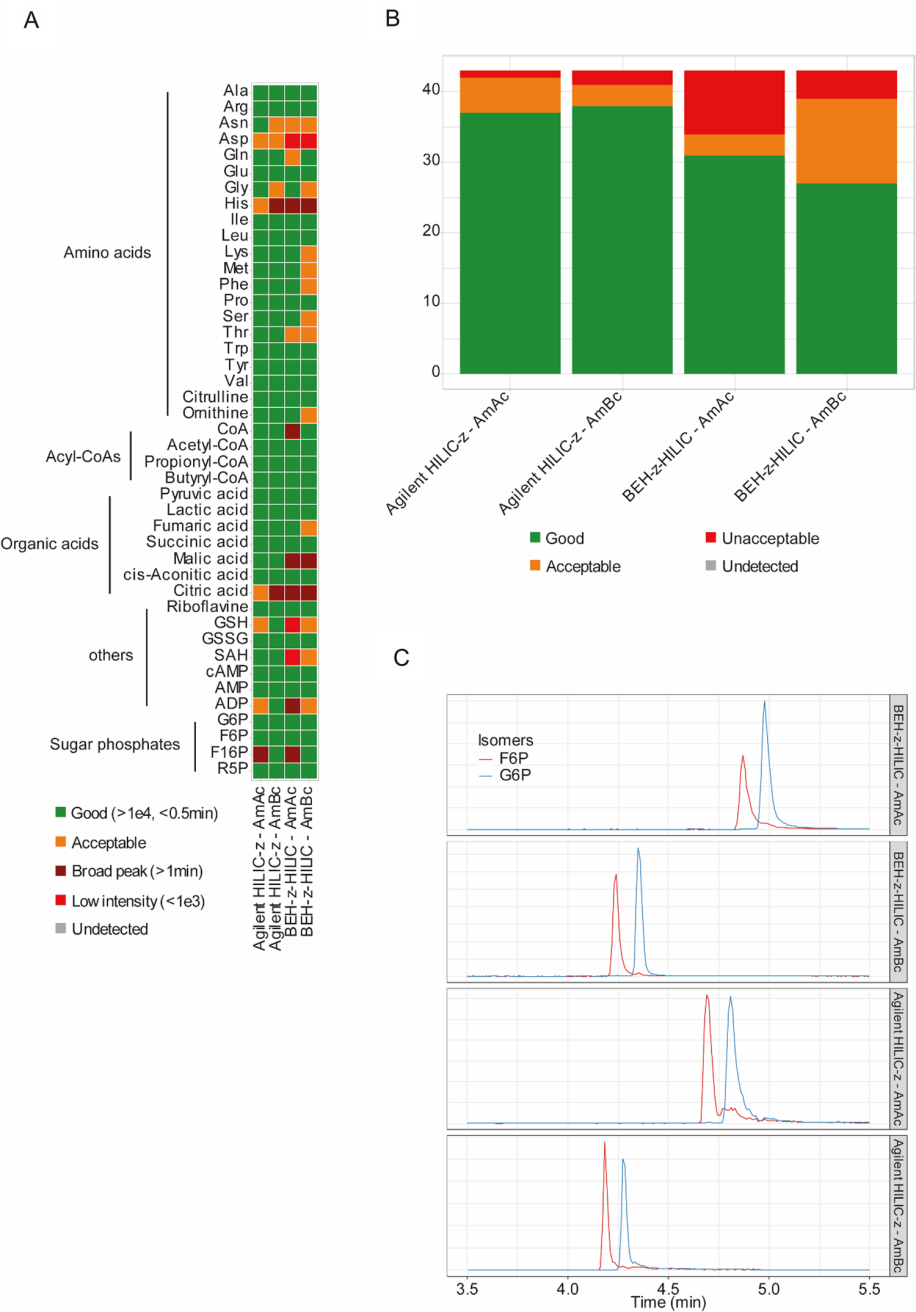
**A** Individual performance of buffers on the two selected columns in negative ionization mode. Scoring is based on peak shapes and intensities. **B** Bar plots representing the number of detected standards analyzed by different candidate columns. **C** Extracted ion chromatograms of the isomeric pair Glucose-6-phosphate/Fructose-6-phosphate using the different buffers and columns in negative ionization mode

After selecting the most promising stationary phases for each ionization mode, the mobile phase compositions were optimized. For positive ionization mode, the recommended buffer system is AmF with low pH (Dai & Hsiao, 2019; Walter et al., 2021). Since the results from the stationary-phase optimization were sufficient, no further optimization was performed. For negative ionization mode, the use of high pH is critical for good retention and peak shapes. The two recommended buffer systems are AmAc (Dai & Hsiao, 2019; Hsiao et al., 2019; Smith et al., 2022; Yannell et al., 2023) and AmBc (Smith et al., 2022; Walter et al., 2021) with high pH. While the first one is often used with the Agilent HILIC-Z, the second one is used with the Waters BEH-Z-HILIC. In the initial test, the buffer substances were directly dissolved in either 100% H_2_O or 100% ACN. However, directly dissolving in pure ACN was cumbersome and could potentially cause problems. To improve buffer dissolution, a 10% H_2_O/90% ACN mixture was used as eluent B in both positive and negative ionization modes. The binary gradient was adapted as a result, starting with 100% B instead of 90% B. Eluent A consisted of 10 mM AmBc or AmAc in water (pH 9), and eluent B consisted of 10 mM AmBc or AmAc in 10% H_2_O/90% ACN (v/v) (pH 9). A summary of the results is presented in Fig. 4. Detected peaks were manually graded as “good” (green) if their height was higher than 1e4 counts, and their width at 5% height was lower than 0.5 min; “broad” (dark read) if their width at 5% height was higher than 1 min; “low intensity” (light red) if their height was lower than 1e3 counts; other detected peaks were marked as “acceptable” (orange).

Overall, most amino acids showed good peak shape for both columns and buffers. In all four setups, phenylalanine was detected with no background noise, unlike in the chosen positive ionization mode column (Agilent HILIC-Z). Glycine and histidine showed multiple peaks, while aspartic acid exhibited a better peak shape with Agilent HILIC-Z and AmAc. CoA and its derivatives showed broader peaks with BEH-Z-HILIC with both buffers. Most organic acids exhibited multiple peaks, making their interpretation more challenging. On the cis-Aconitic acid EIC, it is possible to see the peak of citric acid/isocitric acid with the loss of a molecule of water. Malic acid showed better peak shape with Agilent HILIC-Z, but still had a less well-defined tail; with AmBc, pyruvic acid and lactic acid were detected too close to the void volume (around 0.6 min). While it was not detected during the column choice, GSH was detected but showed multiple peaks across all setups; ADP was not correctly detected with BEH-Z-HILIC and AmAc (broad, poorly defined peaks); SAH peaks were less defined and less intense with BEH-Z-HILIC. The other nucleotides and related molecules showed clear peaks across all setups. Sugar phosphates displayed good peak shape across all four setups, except for F16P, which showed thinner, more intense peaks with AmBc.

Despite having good peak shapes with AmBc, we decided to continue with AmAc. Indeed, due to the heat in an ESI source, AmBc is degraded into carbon dioxide, ammonia, and water, which enhances negative ionization (Nilsson et al., 2024). However, the formation of carbon dioxide leads to protein unfolding and foam production (Hedges et al., 2013). These effects have not yet been described in metabolomics; however, we observed crystallization and column clogging in our LC-MS system when using AmBc. Since some metabolites were detected in both positive and negative ionization modes and exhibited good detection scores in the positive mode (e.g., amino acids or acyl-CoAs), they were not critical for optimizing the negative mobile phases. Despite detecting more metabolites with Agilent HILIC-Z, we chose to use two different columns for positive and negative ionization modes to increase the number of detected metabolites (column complementarity).

The final mobile phases consisted of 10 mM AmF and 0.1% of formic acid in water for eluent A and of 10 mM AmF and 0.1% formic acid in 10% H_2_O/90% ACN for eluent B for positive ionization mode; of 10 mM AmAc and 0.05% ammonium hydroxide 28% in water for eluent A and of 10 mM AmAc and 0.05% ammonium hydroxide 28% in 10% H_2_O/90% ACN for eluent B for negative ionization mode. The final columns were Agilent HILIC-Z for positive-ion mode and BEH-Z-HILIC for negative-ion mode. Finally, the gradient was shortened using the optimal columns and mobile phases. The final chromatographic gradient for both modes was as follows: 0 min, 100% B; 2.0 min, 100% B; 7.5 min, 10% B; 9.0 min, 10% B; 10 min, 90% B; 15 min, 90% B.

During optimization of the negative-ionization mode, we observed a shift in retention time during overnight runs (exceeding 6 h). This is potentially caused by the leakage of ions and salts from borosilicate bottles into the eluents over time (Serafimov et al., 2024). The use of PFA or FEP bottles as eluent bottles improves HILIC separation performance by eliminating retention time shifts (Serafimov et al., 2024; Yannell et al., 2023). In the final method, the usual borosilicate bottles used for the eluents were replaced with Nalgene^®^ FEP bottles, which eliminates the retention time shift.

### Sample preparation optimization

After selecting an appropriate extraction solvent and developing a suitable HILIC method, we aimed to further optimize metabolite extraction from BµS. Following the first experiment on DBS for RPLC-MS, a second experiment was performed using 20 µL of DBS and VAMS for both RPLC-MS and HILIC-MS. The previously chosen extraction solvent, 20% H_2_O/80% MeOH (v/v), was tested both with and without a rehydration step. The rehydration was achieved by adding 80 µL of water, followed by 320 µL of MeOH in a second step. In total, 400 µL of solvent was added to the devices. The samples were sonicated in an ice bath for 10 min and centrifuged at 14,000 rpm for 10 min in a cold room (4–8 °C) after the rehydration step and after solvent addition. Four aliquots of the supernatant were collected, dried under nitrogen, and stored at −80 °C prior to LC-MS analysis. On the day of the analysis, aliquots for RPLC-MS were reconstituted in 40 µL of 80% H_2_O/20% ACN (v/v) and aliquots for HILIC-MS were reconstituted in 40 µL of 10% H_2_O/90% ACN (v/v), sonicated for 5 min, and centrifuged for 5 min at 2,650 rpm.

**Fig. 5:**
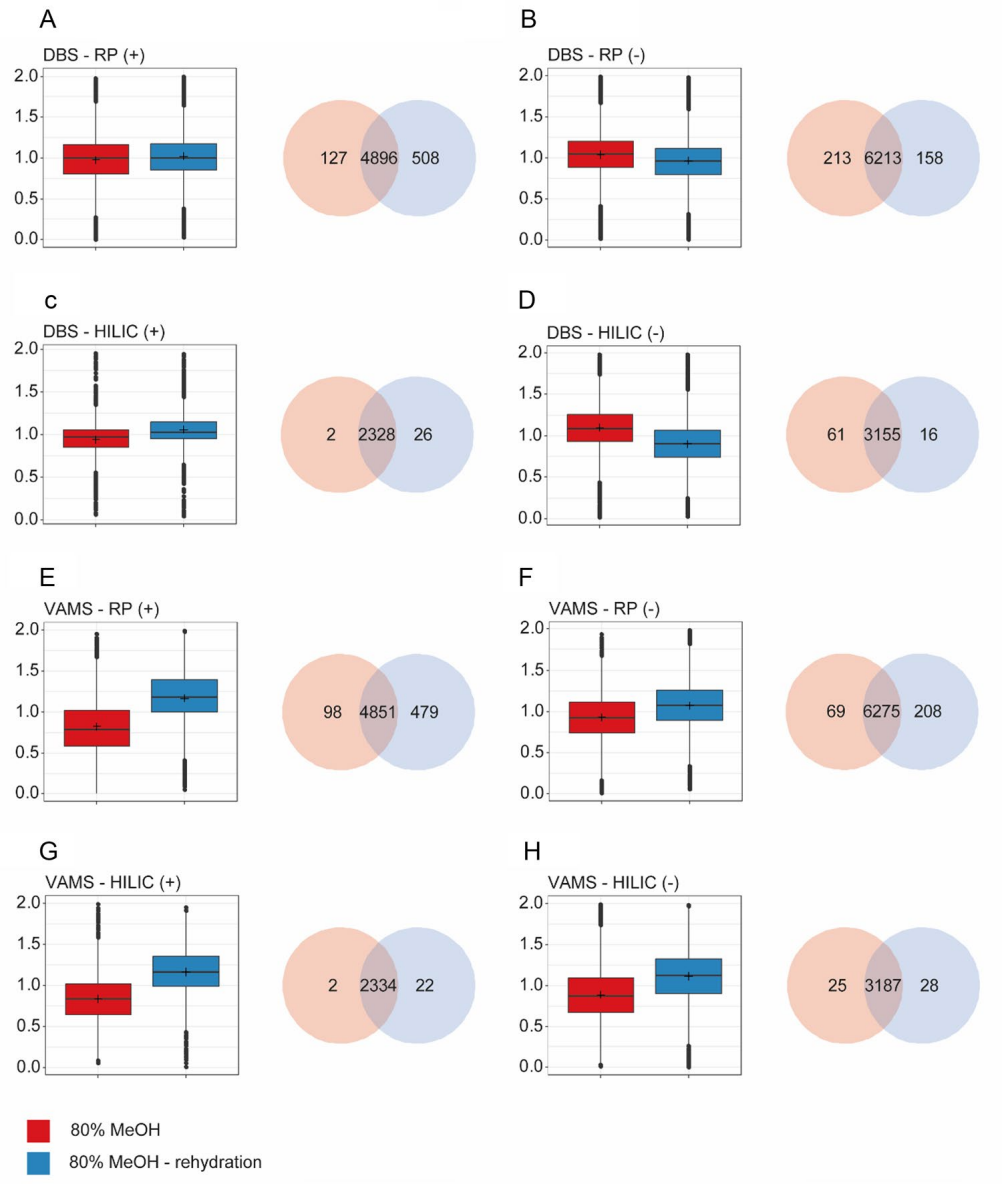
**A**–**H** Boxplots comparing the ratio of peak’s area over average peak’s area for extraction with 80% MeOH applied directly or after rehydration as well as Venn diagram showing the overlap. The respective BµS and analysis method are indicated in the figure

Data were processed with mzmine 4.5 and evaluated based on the number of detected features and their overlap across the different extraction procedures (Fig. 5A–H). As an additional differentiator between the extraction procedures, we compared the intensities of the detected features (Fig. 5A–H). Generally, most features are found in both extraction procedures, and both yield very similar results across the four analysis modes. On average, most features were detected using a 20% H_2_O/80% MeOH (v/v) solution with a rehydration step. We have calculated a peak-area factor for each solvent mixture and compared it to the average across all solvent mixtures. In DBS samples, a 20% H_2_O/80% MeOH (v/v) solution with a rehydration step yielded slightly higher values in the positive ionization mode but slightly lower values in the negative ionization mode. In VAMS samples, a 20% H_2_O/80% MeOH (v/v) solution with a rehydration step yielded slightly higher values in all analysis modes. Since DBS is not a quantitative BµS, and both extraction procedures yielded similar results for both devices, we chose the final extraction procedure as the one that works best for VAMS, since it is a quantitative device. For later application studies, the samples will first be rehydrated, then extracted with methanol.

Rehydration has been used previously in targeted workflows (Couacault et al., 2024; Londhe & Rajadhyaksha, 2020); this study represents a comprehensive evaluation of its value for non-targeted analysis using different chromatographic methods. So far, most methods for extracting BµS in non-targeted metabolomics use direct solvent extraction (Couacault et al., 2024). Though rehydration increases the number of detected features, it also increases the risk of extracting more proteins from the DBS and VAMS samples, which can lead to greater ion suppression and/or column or MS degradation. However, in our application, the ratios of aqueous to organic solvent are similar to those used in typical plasma extraction protocols in metabolomics. Therefore, suppression and degradation effects might be comparable, although no systematic study on this aspect has been performed.

## Metabolite coverage of final methods

Using our final extraction and separation methods, we aimed to evaluate their potential for disease diagnosis by assessing metabolite coverage. BµS are already used in disease detection, such as newborn screening. We highlight a few selected metabolites related to newborn screening that can be detected and annotated with our workflow (Table 1), based on literature (Gelb et al., 2022; Therrell et al., 2024) and governmental recommendations (Health Resources and Services Administration, 2023; National Health Service, 2025). Annotations were performed using mzmine 4.5 with in-house and open libraries, such as those available from GNPS or MassBank. Metabolic disorders covered by newborn screening are primarily linked to amino acid, acylcarnitine, acyl-CoA, and organic acid levels (Therrell et al., 2024). While newborn screening is typically conducted using targeted methods, our methodology also allows us to screen for other abnormalities. However, it is essential to note that, for a comprehensive overview, all methods must be combined. For example, while the RPLC-MS method was very good at separating different C5 acylcarnitines, free carnitine was only detectable with the HILIC-MS positive ionization method, as it elutes in the void volume of the RPLC-MS. Additionally, to the metabolites measured in newborn screening, we added a few selected examples of health-related metabolites that could be annotated with high confidence to showcase the coverage.

**Table 1:** Metabolites detectable with our workflow related to different diseases. “Lvl 1” is detected with level one identification (comparison of RT and MS^2^ spectra with in-house library); “Lvl 2” is detected with level two identification (comparison of MS^2^ spectra with in-house and open libraries). “n.d.” is not detected

	Diseases	Metabolites	RPLC (+)	RPLC (−)	HILIC (+)	HILIC (−)
Inborn errors of amino acid metabolism	Phenylketonuria	Phenylalanine	Lvl 2	Lvl 2	Lvl 1	Lvl 1
		Tyrosine	Lvl 2	Lvl 2	Lvl 1	Lvl 1
	Maple syrup urine disease	Leucine	n.d.	n.d.	Lvl 1	Lvl 1
		Isoleucine	n.d.	n.d.	Lvl 1	Lvl 1
	Homocystinuria	Methionine	n.d.	n.d.	Lvl 1	Lvl 1
	Citrullinemia	Citrulline	n.d.	n.d.	Lvl 1	Lvl 1
Inborn errors of fatty acid metabolism	Systemic primary carnitine deficiency	Free carnitine (C0)	n.d.	n.d.	Lvl 1	n.d.
	Medium-chain acyl-CoA dehydrogenase deficiency (MCADD)	Hexanoyl-carnitine (C6)	Lvl 1	n.d.	Lvl 1	n.d.
		Octanoyl-carnitine (C8)	Lvl 1	n.d.	Lvl 1	n.d.
		Decanoyl-carnitine (C10)	Lvl 1	n.d.	Lvl 1	n.d.
	Very Long-chain Acyl-CoA Dehydrogenase Deficiency (VLCADD)	Tetradecenoyl-carnitine (C14:1)	Lvl 1	n.d.	Lvl 1	n.d.
		Dodecenoyl-carnitine (C12:1)	Lvl 2	n.d.	Lvl 1	n.d.
		Other long-chain acylcarnitines (C12 to C18)	Lvl 1	n.d.	Lvl 1	n.d.
Inborn errors of organic acid metabolism	Propionic Acidemia	Propionyl-carnitine (C3)	Lvl 1	n.d.	Lvl 2	n.d.
	Isovaleric acidemia	Isovaleryl-carnitine (C5)	Lvl 1	n.d.	n.d.	n.d.
Additional health related metabolites	Amino acids	Tryptophan	Lvl 1	Lvl 1	Lvl 1	Lvl 1
		Arginine	n.d.	n.d.	Lvl 1	Lvl 1
		Ornithine	n.d.	n.d.	Lvl 1	Lvl 1
		Lysine	n.d.	n.d.	Lvl 1	Lvl 1
		Glutamine	n.d.	n.d.	Lvl 1	Lvl 1
		Glutamate	n.d.	n.d.	Lvl 1	Lvl 1
	Lipids	LPC 14:0	Lvl 2	n.d.	Lvl 2	n.d.
		LPC 18:0	Lvl 2	n.d.	Lvl 2	n.d.
		LPC 18:1	Lvl 2	n.d.	Lvl 2	n.d.
		LPC 20:4	Lvl 2	n.d.	Lvl 2	n.d.
	Bile acids	Taurocholic acid	Lvl 1	Lvl 1	n.d.	Lvl 2
		Cholic acid	Lvl 1	Lvl 1	n.d.	Lvl 2
		Glycocholic acid	Lvl 1	Lvl 1	n.d.	Lvl 2
	Steroids	Cortisol	n.d.	Lvl 2	n.d.	n.d.
	Gut metabolites	Indoxyl sulfate	n.d.	Lvl 2	n.d.	Lvl 2
	Fatty acids	FA 14:0	n.d.	Lvl 2	n.d.	Lvl 2
		FA 16:0	n.d.	Lvl 2	n.d.	Lvl 2
		FA 18:1	n.d.	Lvl 2	n.d.	Lvl 2
		FA 20:4	n.d.	Lvl 2	n.d.	Lvl 2
	Other metabolites	Creatine	n.d.	n.d.	Lvl 2	Lvl 2
		Creatinine	n.d.	n.d.	Lvl 2	Lvl 2
		Hypoxanthine	n.d.	n.d.	n.d.	Lvl 2
		Uric acid	n.d.	Lvl 2	n.d.	Lvl 2
		Hippuric acid	Lvl 1	Lvl 1	Lvl 1	Lvl 1

## Conclusion and perspectives

We aimed to develop a comprehensive extraction method for BµS, including DBS and VAMS. In the case of non-targeted metabolomics analysis, metabolite coverage is a crucial factor. Our initial experiments showed that a 20% H_2_O/80% MeOH (v/v) mixture is a good compromise for detecting many metabolite features, although other extraction solvent mixtures performed only slightly worse. To increase coverage of the detected metabolites, we developed an HILIC-MS method and used it to further optimize the method. The extent to which the metabolite space is expanded by combining RPLC-MS and HILIC-MS remains open. While comparisons on confidently identified metabolites (e.g., on MSI Level 1) are possible, this depends on the individual available reference spectral libraries for the methods. For example, tryptophan can be detected in all methods and annotated to level 1, but many features remain unannotated. Since BµS are often dried, we used a protocol that included rehydration by adding the aqueous part of the extraction solvent first, followed by the organic part after a brief incubation.

Our extraction and LC-MS methodology covered metabolites from many pathways, including amino acids, acylcarnitines, and bile acids. Acylcarnitines, for example, are an important class of metabolites since they are used in newborn screening for the detection of inborn errors of fatty acid and organic acid metabolism. Non-targeted analysis can also perform such screening, since the fold-changes for diagnosis are typically 100-fold or more. However, as more features are detected, second-tier screening can be performed, enabling more detailed analysis. For example, more comprehensive structure elucidation, such as using Electron-Induced Dissociation (EID) (Ramundi & Witting, 2025), can provide deeper insights into the exact inborn error. Likewise, HILIC enables the detection of many polar metabolites, such as amino acids, and can be used for assessing nutritional status, for example.

## Supplementary Information

Below is the link to the electronic supplementary material.

Supplementary Material 1

## Data Availability

No datasets were generated or analysed during the current study.

## Abbreviations

ACN: Acetonitrile
ADP: Adenosine diphosphate
AmAc: Ammonium acetate
AmBc: Ammonium bicarbonate
AmF: Ammonium formate
AMP: A denosine monophosphate
ATP: Adenosine triphosphate
BµS: Blood microsampling
cAMP: Cyclic adenosine monophosphate
CoA: Coenzyme A
DBS: Dried blood spots
EIC: Extracted-ion chromatogram
ESI: Electrospray ionization
EtOH: Ethanol
F16P: Fructose-1,6-phosphate
F6P: Fructose-6-phosphate
G6P: Glucose-6-phosphate
GSH: Glutathione
GSSG: Glutathione disulfide
H_2_O: Water
HILIC: Hydrophilic interaction chromatography
LC: Liquid chromatography
MeOH: Methanol
MS: Mass spectrometry
R5P: Ribose-5-phosphate
RPLC: Reversed-phase liquid chromatography
SAH: S-adenosylhomocysteine
SAM: S-adenosylmethionine
VAMS: Volumetric absorptive microsampling
