# Supplementary material for: RPLC- and HILIC-based non-targeted metabolomics workflow for blood microsamples
Source: Metabolomics. 2026 Feb 9;22(2):23. doi: 10.1007/s11306-026-02402-y (PMC12886230; doi:10.1007/s11306-026-02402-y)
Supplement: Supplementary file 1 — Supplementary Material 1 [file 11306_2026_2402_MOESM1_ESM.docx]

# Supplementary Information

# RPLC- and HILIC-based non-targeted metabolomics workflow for blood microsamples

Pauline Couacault^1^ , Michael Witting^1,2,^*

^1^ Metabolomics and Proteomics Core, Helmholtz Zentrum München, Ingostädter Landstraße 1, 85764 Neuherberg, Germany

^2^ Chair of Analytical Food Chemistry, TUM School of Life Sciences, Technical University of Munich, Maximus-von-Imhof-Forum 2, 85354 Freising-Weihenstephan, Germany

* corresponding author: Dr. Michael Witting, michael.witting@helmholtz-munich.de

ORCiD

Pauline Couacault: 0009-0008-3044-4135

Michael Witting: 0000-0002-1462-4426

#### SI Table S1: Standards used for HILIC method development and optimization

| **Standard** | **Abbreviations** | **Formula** | **Comments** | **Manufacturer** |
| --- | --- | --- | --- | --- |
| Alanine | Ala | C_3_H_7_NO_2_ |  | Sigma-Aldrich  (Sigma-Aldrich Chemie GmbH, Taufkirchen, Germany) |
| Arginine | Arg | C_6_H_14_N_4_O_2_ |  |  |
| Asparagine | Asn | C_4_H_8_N_2_O_3_ |  |  |
| Aspartic acid | Asp | C_4_H_7_NO_4_ |  |  |
| Cadaverine |  | C_5_H_14_N_2_ |  |  |
| Citrulline |  | C_6_H_13_N_3_O_3_ |  |  |
| Cysteine | Cys | C_3_H_7_NO_2_S |  |  |
| Glutamic acid | Gln | C_5_H_9_NO_4_ |  |  |
| Glutamine | Glu | C_5_H_10_N_2_O_3_ |  |  |
| Glycine | Gly | C_2_H_5_NO_2_ |  |  |
| Histidine | His | C_6_H_9_N_3_O_2_ |  |  |
| Isoleucine | Ile | C_6_H_13_NO_2_ |  |  |
| Leucine | Leu | C_6_H_13_NO_2_ |  |  |
| Lysine | Lys | C_6_H_14_N_2_O_2_ |  |  |
| Methionine | Met | C_5_H_11_NO_2_S |  |  |
| Ornithine |  | C_5_H_12_N_2_O_2_ |  |  |
| Phenylalanine | Phe | C_9_H_11_NO_2_ |  |  |
| Proline | Pro | C_5_H_9_NO_2_ |  |  |
| Putrescine |  | C_4_H_12_N_2_ |  |  |
| Serine | Ser | C_3_H_7_NO_3_ |  |  |
| Spermidine |  | C_7_H_19_N_3_ |  |  |
| Spermine |  | C_10_H_26_N_4_ |  |  |
| Threonine | Thr | C_4_H_9_NO_3_ |  |  |
| Tryptophan | Trp | C_11_H_12_N_2_O2 |  |  |
| Tyrosine | Typ | C_9_H_11_NO_3_ |  |  |
| Valine | Val | C_5_H_11_NO_2_ |  |  |
| Acetyl-CoA |  | C_23_H_38_N_7_O_17_P_3_S | TCA Cycle Metabolite Library |  |
| Oxaloacetic acid |  | C_4_H_4_O_5_ |  |  |
| Malic acid |  | C_4_H_6_O_5_ |  |  |
| CoA |  | C_21_H_36_N_7_O_16_P_3_S |  |  |
| Propionyl-CoA |  | C_24_H_40_N_7_O_17_P_3_S |  |  |
| Butyryl-CoA |  | C_25_H_42_N_7_O_17_P_3_S |  |  |
| Lactic acid |  | C_3_H_6_O_3_ |  |  |
| cis-Aconitic acid |  | C_6_H_6_O_6_ |  |  |
| Adenosine monophosphate | AMP | C_10_H_14_N_5_O_7_P |  |  |
| Cyclic adenosine monophosphate | cAMP | C_10_H_12_N_5_O_6_P |  |  |
| Fructose-1,6-bis-Phosphate | F16P | C_6_H_14_O_12_P_2_ |  |  |
| Ribose-5-Phosphate | R5P | C_5_H_11_O_8_P |  |  |
| Glutathione | GSH | C_10_H_17_N_3_O_6_S |  |  |
| Glutathione disulfide | GSSG | C_20_H_32_N_6_O_12_S_2_ |  |  |
| Riboflavine |  | C_17_H_20_N_4_O_6_ |  |  |
| Fumaric acid |  | C_4_H_4_O_4_ | Organic acids kit | Supelco  (Sigma-Aldrich Chemie GmbH, Taufkirchen, Germany) |
| Succinic acid |  | C_4_H_6_O_4_ |  |  |
| Citric acid |  | C_6_H_8_O_7_ |  |  |
| Isocitric acid |  | C_6_H_8_O_7_ |  |  |
| L-Carnitine | C0 | C_7_H_15_NO_3_ | Acylcarnitines Mix 1 |  |
| O-Acetyl-L-carnitine | C2 | C_9_H_17_NO_4_ |  |  |
| Butyryl-L-carnitine | C4 | C_11_H_21_NO_4_ | Acylcarnitines Mix 2 |  |
| Isobutyryl-L-carnitine | iC4 | C_11_H_21_NO_4_ |  |  |
| Valeryl-L-carnitine | C5 | C_12_H_23_NO_4_ |  |  |
| Isovaleryl-L-carnitine | iC5 | C_12_H_23_NO_4_ |  |  |
| Hexanoyl-L-carnitine | C6 | C_13_H_25_NO_4_ |  |  |
| Octanoyl-L-carnitine | C8 | C_15_H_29_NO_4_ |  |  |
| Decanoyl-L-Carnitine | C10 | C_17_H_33_NO_4_ |  |  |
| Lauroyl-L-carnitine | C12 | C_19_H_37_NO_4_ |  |  |
| Myristoyl-L-carnitine | C14 | C_21_H_41_NO_4_ |  |  |
| Stearoyl-L-carnitine | C18 | C_25_H_49_NO_4_ |  |  |
| trans-2-octenoyl-L-carnitine | C8:1 | C_15_H_27_NO_4_ | Acylcarnitines Mix 3 |  |
| trans-2-decenoyl-L-carnitine | C10:1 | C_17_H_31_NO_4_ |  |  |
| trans-2-dodecenoyl-L-carnitine | C12:1 | C_19_H_35_NO_4_ |  |  |
| trans-2-tetradecenoyl-L-carnitine | C14:1 | C_21_H_39_NO_4_ |  |  |
| trans-2-hexadecenoyl-L-carnitine | C16:1 | C_23_H_43_NO_4_ |  |  |
| oleoyl-L-carnitine | C18:1 | C_25_H_47_NO_4_ |  |  |
| cis-cis-5,8-tetradecandienoyl-L-carnitine | C14:2 | C_21_H_37_NO_4_ |  |  |
| cis,cis-9,12-octadecadienoyl-L-carnitine | C18:2 | C_25_H_45_NO_4_ |  |  |
| Isobutyryl-CoA |  | C_25_H_42_N_7_O_17_P_3_S |  | Cayman Chemical  (Biomol GmbH, Hamburg, Germany) |
| Fructose-6-Phosphate | F6P | C_6_H_13_O_9_P |  |  |
| Glucose-6-Phosphate | G6P | C_6_H_13_O_9_P |  |  |
| S-Adenosylhomocysteine | SAH | C_14_H_20_N_6_O_5_S |  |  |
| S-Adenosylmethionine | SAM | C_15_H_23_N_6_O_5_S |  |  |
| Pyruvic acid |  | C_3_H_4_O_3_ |  | Santa Cruz Biotechnology  (Santa Cruz Biotechnology Inc., Heidelberg, Germany) |
| Adenosine diphosphate | ADP | C_10_H_15_N_5_O_10_P_2_ |  |  |
| Adenosine triphosphate | ATP | C_10_H_16_N_5_O_13_P_3_ |  | Carl Roth  (Carl Roth GmbH, Karlsruhe, Germany) |

#### SI Table S2: MS parameters

| **Parameter** | **RPLC-MS positive** | **RPLC-MS negative** | **HILIC-MS positive** | **HILIC-MS negative** |
| --- | --- | --- | --- | --- |
| Method duration | 12.1 min | 12.1 min | 10 min | 10 min |
| **Source and Gas Parameters** | | | | |
| Ion source gas 1 | 45 psi | 45 psi | 45 psi | 45 psi |
| Ion source gas 2 | 45 psi | 45 psi | 45 psi | 45 psi |
| Curtain gas | 40 psi | 40 psi | 40 psi | 40 psi |
| CAD gas | 7 | 7 | 7 | 7 |
| Temperature | 500°C | 500°C | 500°C | 500°C |
| **Experiment** | | | | |
| Experiment | IDA | IDA | IDA | IDA |
| Polarity | Positive | Negative | Positive | Negative |
| Spray voltage | 5500 V | -4500 V | 5500 V | -4500 V |
| **TOF MS** | | | | |
| TOF start mass | 70 Da | 70 Da | 70 Da | 70 Da |
| TOF stop mass | 1500 Da | 1500 Da | 1500 Da | 1500 Da |
| Accumulation time | 0.1 s | 0.1 s | 0.1 s | 0.1 s |
| Declustering potential | 80 V | -80 V | 80 V | -80 V |
| DP spread | 0 V | 0 V | 0 V | 0 V |
| Collision energy | 10 V | -10 V | 10 V | -10 V |
| CE Spread | 0 V | 0 V | 0 V | 0 V |
| **TOF MS - Advanced Experiment Settings** | | | | |
| Time bins to sum | 6 | 6 | 6 | 6 |
| Channel 1 | TRUE | TRUE | TRUE | TRUE |
| Channel 2 | TRUE | TRUE | TRUE | TRUE |
| Channel 3 | TRUE | TRUE | TRUE | TRUE |
| Channel 4 | TRUE | TRUE | TRUE | TRUE |
| Override QJet RF value | FALSE | FALSE | FALSE | FALSE |
| IDA Criteria | Small molecule | Small molecule | Small molecule | Small molecule |
| Maximum Candidate ions | 12 | 12 | 12 | 12 |
| Intensity treshold exceeds | 100 cps | 100 cps | 100 cps | 100 cps |
| Dynamic background subtraction | TRUE | TRUE | TURE | TRUE |
| Exclude former candidate ions | TRUE | TRUE | TRUE | TRUE |
| For | 1 s | 1 s | 1 s | 1 s |
| After | 1 occurrences | 1 occurrences | 1 occurrences | 1 occurrences |
| Exclusion List | TRUE | TRUE | TRUE | TRUE |
| Exclude isotopes +/- | 4 Da | 4 Da | 4 Da | 4 Da |
| Mass tolerance +/- | 50 mDa | 50 mDa | 50 mDa | 50 mDa |
| **TOF MSMS** | | | | |
| Fragmentation mode | CID | CID | CID | CID |
| TOF start mass | 50 Da | 50 Da | 50 Da | 50 Da |
| TOF stop mass | 1500 Da | 1500 Da | 1500 Da | 1500 Da |
| Accumulation time | 0.025 s | 0.025 s | 0.025 s | 0.025 s |
| Declustering potential | 80 V | -80 V | 80 V | -80 V |
| DP Spread | 0 V | 0 V | 0 V | 0 V |
| Zeno pulsing | TRUE | TRUE | TRUE | TRUE |
| Q1 resolution | Unit | Unit | Unit | Unit |
| Collision energy | 35 V | -35 V | 35 V | -35 V |
| CE spread | 15 V | 15 V | 15 V | 15 V |
| **TOF MSMS - Advanced Experiment Settings** | | | | |
| Time bins to sum | 6 | 6 | 6 | 6 |
| Channel 1 | TRUE | TRUE | TRUE | TRUE |
| Channel 2 | TRUE | TRUE | TRUE | TRUE |
| Channel 3 | TRUE | TRUE | TRUE | TRUE |
| Channel 4 | TRUE | TRUE | TRUE | TRUE |
| Zeno treshold | 80000 cps | 80000 cps | 80000 cps | 80000 cps |
| Override QJet RF value | FALSE | FALSE | FALSE | FALSE |

#### SI Table S3: RPLC parameters (Sciex ExionLC AD)

| **Parameter** | **Value** |
| --- | --- |
| **AD Pump** | |
| Stop time | 15.00 min |
| Flow | 0.500 mL/min |
| A.Conc | 95.0% |
| B.Conc | 5.0% |
| B.Curve | 0 |
| Pressure limits Minimum | 20 bar |
| Pressure limits Maximum | 1100 bar |
| Gradient | Advanced |
|  | 1.50 min 0.500 mL/min 95.0%A 5.0%B |
|  | 10.00 min 0.500 mL/min 0.1%A 99.9%B |
|  | 12.00 min 0.500 mL/min 0.1%A 99.9%B |
|  | 12.10 min 0.500 mL/min 95.0%A 5.0%B |
| **Solenoid valve** | |
| Pump A: Solvent Valve | A-A-A |
| **Compressibility settings** | |
| Compressibility settings | TRUE |
| Mobile Phase A | Water 0.45/GPa |
| Mobile Phase B | Acetonitrile 1.20/GPa |
| **AD Autosampler** | |
| Sampling speed | 5.0 µL/s |
| Cooler temperature | TRUE, 5°C |
| **AD Autosampler - Rinse settings** | |
| Rinse type | Internal & external |
| Rinse mode | Before and after aspiration, Dip time:0s |
| Rinse pumd method | Rinse port only, Time: 2s |
| Rinse port liquid | R1 |
| Rinsing speed | 35 µL/s |
| Rinse volme | 500 µL |
| Measuring line purge volume | 600 µL |
| Rinsing start time | Specify start time 12.1 min |
| Rinse sequence | R2 --> R0 |

#### SI Table S4: HILIC parameters (Agilent 1290 Infinity II Bio)

| **Parameter** | **Value (Positive)** | **Value (Negative)** |
| --- | --- | --- |
| **Binary Pump** | | |
| Stop time | 15.00 min | 15.00 min |
| Flow | 0.500 mL/min | 0.500 mL/min |
| A.Conc | 0.0% | 0.0% |
| B.Conc | 100.0% | 100.0% |
| B.Curve | 0 | 0 |
| Pressure limits Minimum | 20 bar | 20 bar |
| Pressure limits Maximum | 1300 bar | 1300 bar |
| Gradient | Advanced | Advanced |
|  | 2.00 min 0.500 mL/min 0%A 100%B | 2.00 min 0.500 mL/min 0%A 100%B |
|  | 7.50 min 0.500 mL/min 90%A 10%B | 7.50 min 0.500 mL/min 90%A 10%B |
|  | 9.00 min 0.500 mL/min 90%A 10%B | 9.00 min 0.500 mL/min 90%A 10%B |
|  | 10 min 0.500 mL/min 0%A 100%B | 10 min 0.500 mL/min 0%A 100%B |
| Use Solvent Types | TRUE | TRUE |
| Mobile Phase A | Water + 10mM AF + 0.1% FA | Water + 10mM AA, pH9 |
| Mobile Phase B | Acetonitrile/Water (90:10)  + 10mM Ammo Formate  + 0.1% Formic Acid | Acetonitrile/Water (90:10)  + 10mM Ammo Acetate, pH9 |
| **Multisampler** | | |
| Draw speed | 100 µL/min | 100 µL/min |
| Eject speed | 100 µL/min | 100 µL/min |
| Wait time after drawing | 1.2 s | 1.2 s |
| Injection Volume | 5 µL | 5 µL |
| Cooler temperature | TRUE, 5°C | TRUE, 5°C |
| **Rinse settings** | | |
| Needle Wash Mode | Flush Port | Flush Port |
| Mode | Standard Wash | Standard Wash |
| Duration | 3 s | 3 s |
| **Column Comp.** | | |
| Oven temperature | 40°C | 40°C |
